# Unleashing rhizobacteria for sustainable soil remediation: PGPR roles in heavy metal tolerance, detoxification, and plant productivity

**DOI:** 10.3389/fmicb.2025.1662000

**Published:** 2025-09-09

**Authors:** Priya Kaushal, Aparna Maitra Pati

**Affiliations:** ^1^Biotechnology Division, CSIR-Institute of Himalayan Bioresource Technology, Palampur, HP, India; ^2^Department of Biotechnology, Guru Nanak Dev University, Amritsar, Punjab, India; ^3^Academy of Scientific and Innovative Research (AcSIR), Ghaziabad, India

**Keywords:** bioremediation, heavy metals, plant growth promoting rhizobacteria, sustainable agriculture, crop productivity

## Abstract

The United Nations Food and Agriculture Organization (FAO) has projected that by 2050, nearly 90% of the world's soil resources will be at risk due to factors such as erosion, overuse of agrochemicals, and industrial pollution. As soil sustains over 95% of the global food supply, such degradation poses a critical threat to food security and ecosystem stability. Among the myriad environmental pollutants, heavy metals (HMs) like arsenic (As), lead (Pb), cadmium (Cd), and chromium (Cr) stand out as insidious threats to the environment. Addressing this issue demands the adoption of eco-friendly and sustainable remediation strategies. Microbial-assisted bioremediation particularly involving plant growth-promoting rhizobacteria (PGPRs) has emerged as a promising approach to enhance HMs detoxification while supporting plant health and soil recovery. In this review, we compile and critically evaluate current literature on PGPR-mediated bioremediation, with a focus on mechanisms of HMs tolerance and detoxification, the impact of PGPRs on soil health, and their role in promoting plant growth in contaminated environments. Overall, aims of the study is to provide a holistic understanding of microbial strategies for managing HMs pollution in soil–plant systems, offering a sustainable path forward for agricultural productivity and environmental restoration.

## 1 Introduction

Heavy metals (HMs) are metallic elements characterized by relative atomic mass >40, specific density of 5 g/cm^3^, and specific gravity 4–5 times higher than water (Timothy and Williams, 2019). These are natural components of environment but their toxicity, bioaccumulation, long-term stability, non-biodegradable, and persistent nature poses a significant environmental concern. In addition to natural sources, anthropogenic activities like industrial waste, sewage disposal, chemical fertilizers, pesticides, insecticides, agricultural runoff, ore mining, smelting, fuels, and electronic waste substantially contribute to HMs contamination (Ali et al., 2021). According to the Agency for Toxic Substances and Disease Registry (ATSDR), As, Pb, Cd, and Cr are among the highly toxic HMs, ranking 1st, 2nd, 7th, and 17th position on the list of hazardous substances. Globally, the estimated annual production of these metals is 36,000–45,000 tons for As, 4.8 × 10^6^ tons for Pb, 20,000–24,000 tons for Cd, and 18 × 10^6^-30 × 10^6^ tons for Cr (Rahman and Singh, 2019). HMs negatively impacts soil physicochemical properties, ultimately compromising plant health and reducing crop yield. (Angon et al., 2024; Devi et al., 2022; Nyiramigisha and Komariah, 2021; Vasilachi et al., 2023). In addition, the transfer of HMs through trophic levels—from contaminated water and soil to food crops—poses significant health risks to humans. This bioaccumulation can disrupt the normal functioning of vital systems, including the central nervous, respiratory, reproductive, and gastrointestinal systems, as well as adversely affect liver and cardiac function (Alipour et al., 2024; Waqas et al., 2024). To address this issue, various chemical and physical methods such as precipitation, ion exchange, chemical leaching, oxidation-reduction, immobilization, electro-kinetics, and vitrification have been used for soil remediation, but they often generate toxic sludge, and disrupt soil quality (Sharma et al., 2018; Kumar et al., 2020). Due to these drawbacks, research has increasingly shifted towards microbial-based bioremediation approaches, which are recognized for their cost-effectiveness, environmental sustainability, and minimal ecological disruption (Firincá et al., 2025).

Microorganisms are ubiquitous in nature and constitute a fundamental component of the ecosystem, engaging in intricate interactions with soil chemical contaminants (Abdu et al., 2017). The application of rhizosphere-associated microbes as soil amendments has demonstrated significant potential in enhancing plant growth, primarily through improvements in soil physicochemical properties and root system architecture. Numerous PGPRs, including members of the genera *Bacillus, Serratia, Arthrobacter, Pseudomonas, Rhodococcus, Enterobacter, Acinetobacter*, and *Ochrobactrum*, have been widely reported for their dual functionality—promoting plant growth and facilitating HMs detoxification (Abdelkrim et al., 2018; Gulzar and Mazumder, 2022; Kaushal et al., 2023). While their role in mitigating HMs-induced phytotoxicity is well-studied, practical insights into their impact on long-term soil health remain limited. Similarly, plant-focused studies often emphasize early-stage responses, with insufficient insights in understanding whole-plant physiology, yield performance, and molecular mechanisms under HMs stress. Therefore, the present review aims to comprehensively evaluate the potential of PGPRs in enhancing soil health, and to elucidate interactive roles in mitigating heavy metal stress in soil–plant systems.

## 2 HMs tolerance and their mechanism of detoxification by PGPRs

**Figure 1:**
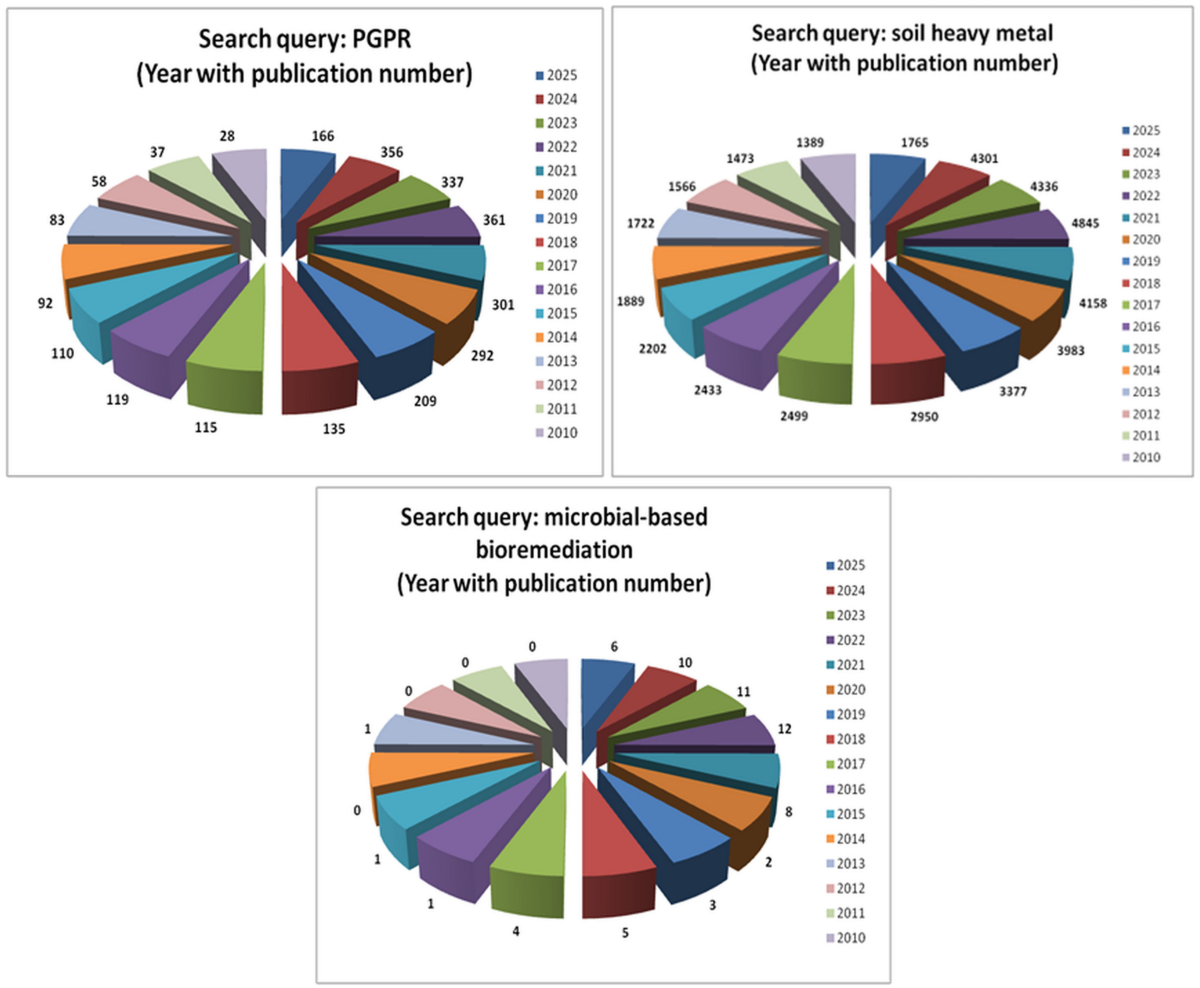
Trends in the number of publications related to PGPR and soil heavy metal bioremediation.

PGPRs employ a multitude of mechanisms to mediate HMs detoxification including biosorption, bioaccumulation, biodegradation, biotransformation, precipitation, complexation, redox reactions, metal chelation via siderophores, complexation through exopolysaccharides (EPS) production, active efflux, and influx transporters (Gupta et al., 2023). Numerous studies, as shown in Table 1, and Figure 1 have documented on PGPRs, HMs tolerance, bioaccumulation potential of various PGPRs strains, and microbe-based bioremediation, the molecular mechanism underpinning these processes—particularly at the genomic and transcriptomic levels—remains relatively underexplored. To bridge this gap, this section critically highlights recent investigations that delve into the molecular-level insights of PGPRs, with a focus on the identification of key metal resistance genes, operons, transport systems, and regulatory elements that orchestrate their survival and function in metal-contaminated environments.

**Table 1:** List of HMs tolerance and bioaccumulation ability of previously reported PGPR strains.

**PGPRs**	**HMs Used**	**HMs tolerance range**	**HMs bioaccumulation efficiency**	**Time of HMs bioaccumulation**	**References**
*Pseudomonas aeruginosa*	Cadmium Chloride	2,200 ppm	**–**	**–**	Lin et al., 2016
	Lead	1,200 ppm	**–**	**–**	
*Brevundimonas diminuta*	Sodium arsenate As (V)	150 mM	20–21%	24–36 h	Singh et al., 2016
	Sodium arsenite As (III)	20 mM	14–38%		
*Enterobacter* sp.	Cadmium	3,000 μg/mL	73%	72 h	Mitra et al., 2018
	Lead	2,500 μg/mL	–	**–**	
	Arsenic	1,050 μg/mL	–	**–**	
*Bacillus aryabhattai*	As (V)	100 mM	41%	24–36 h	Ghosh et al., 2018
	As (III)	20 mM	26%		
*Cellulosimicrobium* sp.	Potassium chromate	800 mg/L	Reduced 100 mg/L	48 h	Tirry et al., 2018
*Bacillus subtilis* BM2	Lead acetate	2,000 μg/mL	**–**	**–**	Rizvi et al., 2019
*Bacillus flexus*	As (V), As (III)	150 and 70 mmol L^−1^	Above 80% [As (V)]	72 h	Marwa et al., 2019
*Acinetobacter junii*			60% [As (V)]		
*Bacillus cereus* 2M1	Lead acetate	800 ppm	–	–	Abdullahi et al., 2020
*Bacillus cereus* 3M1					
*Bacillus pseudomycoides* 3M3	Lead acetate, Cadmium nitrate tetrahydrate	800 ppm (Pb), 300 ppm (Cd)			
*Enterobacter tabaci* 4M9	Cadmium nitrate tetrahydrate	300 ppm			
*Pseudomonas plecoglossicida* 6M2	Sodium arsenite	1,700 ppm			
*Bacillus tropicus*	Lead nitrate	1,400 ppm	–	–	Efe, 2020
*Stenotrophomonas* sp.	Lead (II) chloride, Potassium chromate	500–1,000 μg/mL (Cr) and 1,000–1,600 μg/mL (Pb)	68.54% (Cr), and 85.3% (Pb)	24–72 h	Aslam et al., 2020
*Klebsiella pneumoniae*			65.98% (Cr), and 65.85% (Pb)		
*Staphylococcus* sp.			71.45 % (Cr), and 65.85 % (Pb)		
*Bacillus cereus* BPS-9	Lead nitrate	2,400 ppm	79.26 %	72 h	Sharma and Shukla, 2021
	Cadmium Chloride	500 ppm	–	–	
	Chromium	600 ppm	–	–	
*Pseudomonas* sp.	Lead nitrate	Above 50 mg/mL	Above 80 %	40 h	Vélez et al., 2021
*Bacillus subtilis*	Potassium dichromate	25 ppm	–	–	Ilyas et al., 2022
*Enterobacter cloacae*	Cadmium chloride	4,000 μg/mL	72.11 %	72 h	Ghosh et al., 2022
*Bacillus subtilis*	Cadmium nitrate	100 ppm	92.3 %	1–144 h	Rocco et al., 2023
	Lead acetate	500 ppm	100 %		
*Bacillus velezensis* QZG6	Cadmium chloride	100–400 μM	–	–	Chen et al., 2024
*Bacillus altitudinis*	Lead acetate	15 mM	96 %	48 h	Kaushal and Pati, 2024
*Bacillus cereus* NM8	Potassium dichromate	100 μg/mL	–	–	Malik et al., 2024
*Bacillus subtilis* NM28					
*Bacillus paramycoides*	Cadmium chloride	2,000 ppm	81.79 %	72 h	Zainab et al., 2024
*Bacillus tequilensis*			83.78%		
*Stenotrophomonas maltophilia*	Lead nitrate	18 mM	78.4% (removal efficiency)	70 min	Farooq et al., 2025
*Bacillus altitudinis* 41KF2b	Chromium	100 ppm	51.0%	120 h	Hamed et al., 2025
*Bacillus tropicus*			45.1%		
*Providencia rettgri*			19.1%		
*Pseudomonas* sp.	Lead	500 ppm	61.3%	72 h	Jabborova et al., 2025

Xu et al. (2018) work demonstrated remarkable tolerance of *Arthrobacter* sp. PGP41, to 1.58 mM Cd by secreting various low-molecular-weight organic acids, including oxalic, tartaric, formic, malic, citric, and succinic acids, which likely contributed to Cd chelation and detoxification through metal complexation. In a genomic study by Luziatelli et al. (2020), *Pantoea agglomerans* C1 strain exhibited high tolerance to As (V) up to 100 mM. Genomic analysis revealed the presence of key arsenic resistance determinants including arsenite efflux transporter (*arsB*), arsenate reductase (*arsC*), arsenite efflux pump (*acr3*), and transcriptional regulator (*arsR*), supporting a genetically encoded mechanism for arsenic detoxification. Liaquat et al. (2020) reported that *Stenotrophomonas maltophilia* SY-2 strain tolerated 1.0 mM Cd and exhibited a Cd biosorption capacity of 35.7% within 24 h. The study also noted enhanced siderophore production under Cd stress, and Fourier Transform Infrared (FTIR) spectroscopy confirmed the involvement of functional groups such as carboxyl, amide, and phosphate moieties in metal ion binding on the bacterial surface.

Furthermore, Chlebek et al. (2021) study elucidated that *Pseudomonas qingdaonensis* ZCR6 strain tolerated 5 mM Cd and genome-wide analysis confirmed the presence of cobalt-zinc-cadmium resistance genes (*czcA, czcB*, and *czcC*) that helps in the transport of divalent cations, and detoxification of Zn^2+^, Co^2+^, and Cd^2+^. Moreover, Afridi et al. (2021) reported that *Kocuria rhizophila* (14ASP) endophytic strain tolerated Cr (500 ppm), Pb (200 ppm), and Cd (50 ppm), following 6–7 days of incubation. Genomic insights revealed the presence of chromate transporter (*chrA*), lead resistance (*pbrA*), and cadmium resistance (*cadB*) genes, indicating a broad-spectrum HMs resistance capability.

More recently, Tatung and Deb (2024) reported two promising isolates—*Bacillus cereus* (TSU3) and *Pseudomonas koreensis* (TSU7), that could tolerate 8,070 μg/mL Cr and 570 μg/mL Cd, respectively. Interestingly, Herrera-Calderon et al. (2024) also reported that *Bacillus velezensis* (C3-3) and *Cytobacillus gottheilii* (T106) showed resistance to 5 mM of Cd due to the presence of Co/Zn/Cd genes in its genome (*czcA, cusA, cnrA, czcD, zitB)*.

In conclusion, these investigations provide critical insights into the molecular frameworks that underpin PGPR-mediated HMs detoxification. Such knowledge not only advances our understanding of microbial adaptation and resistance but also lays the foundation for restoring soil health and promoting sustainable plant growth in HMs-stressed environments.

## 3 Effect of PGPRs on HMs contaminated soil health

Soil is a fundamental component of terrestrial ecosystems and plays a pivotal role in sustaining agricultural productivity. However, excessive HMs contamination significantly disrupts soil structure, pH, porosity, density, texture, electrical conductivity, and water holding capacity. It also impairs biological functions including litter decomposition, organic matter stability, nutrients availability, carbon mineralization, nitrogen transformation processes, enzymatic activity, and the microbial diversity (Chu, 2018; Lwin et al., 2018; Srivastava et al., 2017). In addition to this, different PGPRs have been reported for their role in HMs bioaccumulation, improving soil quality, and enzyme activities that helps in nutrient recycling, and plant growth. Despite these findings, the important gaps remain in understanding the role of PGPRs in restoring soil health under HM stress.

A notable study by Abdelkrim et al. (2018) highlights the effect of PGPRs consortia (*Rhizobium leguminosarum* (M5) + *Bacillus simplex* + *Luteibacter* sp. + *Variovorax* sp.) (I1) and (*R. leguminosarum* (M5) + *Pseudomonas fluorescens* (K23) + *Luteibacter* sp. + *Variovorax* sp.) (I5) on Pb, and Cd polluted soil of *L. sativus* plots. The results showed that I5 inoculum treatment reduces total Pb, and Cd by 46%, and 61%, respectively as compared to uninoculated soil. Moreover, I5 inoculation also significantly enhanced total nitrogen content (N), available phosphorus (P), β-glucosidase, urease and alkaline phosphatase of soil by 35, 100, 16, 12, and 32%, respectively, relative to uninoculated soil. Additionally, He et al. (2020) also reported that *Bacillus* sp. QX8 and QX13 treatment under Pb, and Cd contamination significantly improved the soil acid phosphatase, and urease activity by 23, and 22%, respectively as compared to untreated soil. Furthermore, Silva et al. (2021) findings showed that PGPRs consortia treatment increased soil respiration rate, and microbial biomass C (MBC) under 320 mg/kg Cr stress. Complementary findings were reported by Liu et al. (2022), who evaluated the efficacy of *Bacillus* sp. ZC3-2-1 in Cd-contaminated soils. The strain decreased bioavailable Cd levels by 39.3% and significantly boosted protease and alkaline phosphatase activities by 45.8, and 6.4%, respectively in the soil. Moreover, treatment with ZC3-2-1 also led to a marked increase in bacterial alpha diversity and the relative abundance of beneficial taxa such as *Actinobacteria, Proteobacteria*, and *Bacteroidetes*, that helps in nitrogen and phosphorus cycling in HMs contaminated soil. Further, Haroun et al. (2023) demonstrated the remediation potential of a biofertilizer formulation comprising *Pseudomonas aeruginosa* and *Bacillus firmus* in HM-polluted soils. The biofertilizer significantly improved key soil physicochemical properties, including available N, P, K, and organic matter content. Enzymatic activities of dehydrogenase, alkaline phosphatase, and β-D-glucosidase were also substantially enhanced in biofertilizer-treated soils compared to control plots. Recent study by Nie et al. (2025) showed that Pb resistant *Pseudomonas* sp. and *Bacillus* sp. treatment significantly decreased the Pb content, improved the available P, and K in the Pb contaminated rhizosphere soil.

Collectively, these studies underscore the promising role of PGPRs in mitigating heavy metal toxicity, enhancing nutrient cycling, and restoring soil biological activity, thereby contributing to sustainable soil health management in contaminated agroecosystems.

## 4 Effect of PGPRs on plant growth under HMs stress

**Figure 2:**
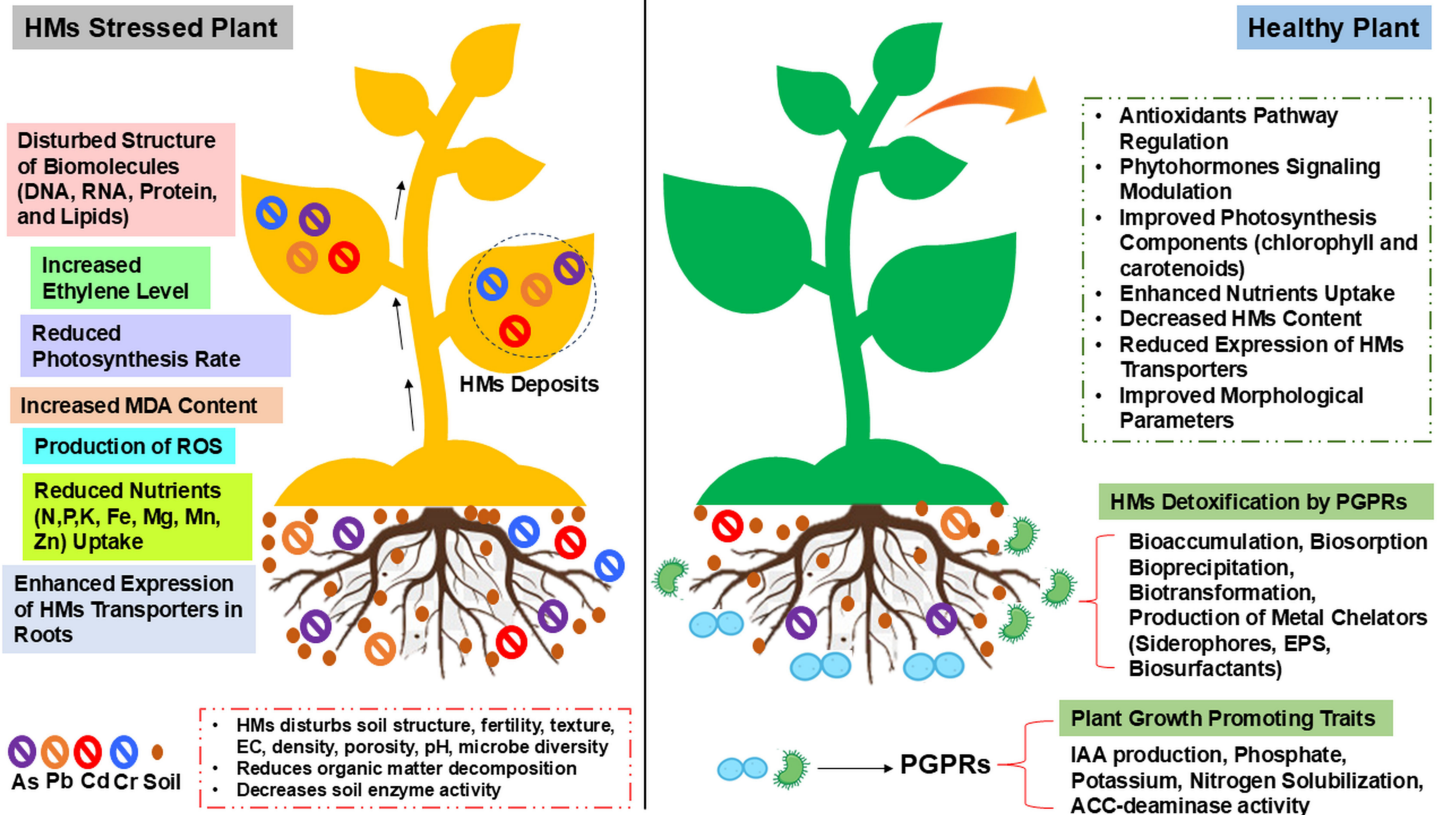
Effect of PGPRs on plant growth and development during HMs stress.

HMs accumulation in soil adversely impairs plant growth and development by disrupting key morpho-physiological, biochemical, and molecular processes. Toxic metal ions interfere with root architecture, reduce seed germination rates, induce nutrient imbalances, and cause chlorosis and impaired photosynthetic efficiency. At the cellular level, elevated HMs stress promotes excessive generation of reactive oxygen species (ROS), leading to oxidative damage. This includes lipid peroxidation of cell membranes, degradation of structural and functional proteins, and alterations in DNA integrity. The schematic representation of these pathways is presented in Figure 2. Collectively, these effects compromise plant vitality and significantly reduce crop productivity (Angon et al., 2024). However, PGPRs have been extensively reported to mitigate HMs toxicity by reducing their bioavailability. In addition to metal immobilization, PGPRs also enhance plant stress resilience by modulating antioxidant defense systems, regulating stress-responsive signaling pathways, and promoting growth by facilitating nutrient availability, phytohormone, exopolysaccharide (EPS) production, and ACC-deaminase activity (Dey et al., 2022; Wang et al., 2024). In this context, key studies elucidating the molecular and physiological pathways targeted by PGPRs in different plants under HM stress are discussed in the following sections and summarized in Table 2.

**Table 2:** Effect of PGPRs application on plant growth under HMs stress.

**PGPRs**	**Plant**	**HMs contamination**	**Effect of PGPR on HMs uptake in plants**	**Effect of PGPR inoculation on plant growth**	**References**
*Pseudomonas gessardii*	*Helianthus annus*	600 mg/kg Pb (NO_3_)_2_	62.42% reduction in Pb translocation from root to shoot	Chlorophyll a, b, and total chlorophyll content, grain weight increased by 47.36%, 37.30%, 44.02%, 15.93%	Raza Altaf et al., (2021)
*Pseudomonas fluorescens, Pseudomonas putida*, and *Bacillus safensis*	*Brassica napus* and *Trifolium repens*	400, 800, and 1,200 mg/kg Pb	–	Increased number of pods per plant, chlorophyll a, b, carotenoid, proline content, SOD, and GR activity	Shah et al., (2020)
*Bacillus gibsonii*, and *Bacillus* xiamenensis	*Sesbania sesban* L.	Cd (5.1 ppm), Cr (26.4 ppm), and Pb (42 ppm)	Increased Pb, and Cr uptake	Increased chlorophyll, carotenoid content, SOD, POD enzymatic actiivty	Zainab et al., (2021)
*Pseudomonas* sp.	*Anethum graveolens* L.	0, 100, 400 mg/kg Pb (NO_3_)_2_	Reduced Pb uptake	Total carbohydrate, proline, chlorophyll content, CAT, POD activity was enhanced	Rahbari et al., (2021)
*Morganella morganii* (ABT3, and ABT9)	*Arabidopsis thaliana*	1.5, and 2.5 mM Pb (NO_3_)_2_	–	Increased shoot, root fresh, dry weight, length, chlorophyll content, quantum yield, SOD, POD, and CAT activity	Naqqash et al., (2022)
*Enterobacter cloacae*	*Oryza sativa* (IET-15,191 Cd sensitive)	0–400 μg/mL	Reduced Cd content by 95 μg/g fresh weight	Improved morphological parameters, total sugar, protein, proline, chlorophyll content, α-amylase, protease, and antioxidant activity. Significant reduction in MDA, and ethylene level in plants	Ghosh et al., 2022
Consortia^*^	*Oryza sativa* (IR64 Variety)	800 mg/L Pb	45% reduction in Pb content	Chlorophyll content increased by 25%, MDA, and ROS content reduced by 50%, and 13%	Ubaidillah et al., 2023
*Pseudomonas fluorescence*	*Cicer arietinum* L.	400 μg/kg Cd	37% decrease in Cd deposition in roots	Increased seed germination (10%), root length (25%), plant length (26.5%), chlorophyll a (34%), b (29%), carotenoid (41%), seed protein (20%), content, and seed yield (26%),	Syed et al., 2023
S2, S5, and S10 Pb tolerant PGPR	*Brassica juncea*	300, 600, 900 mg/kg Pb	Ameliorated Pb uptake in plant by 9.2%. 26% reduction of Pb in seeds	Improved agronomic growth parameters (number of pods, seeds, yield per plant), chlorophyll a, b, carotenoid, proline content, SOD, CAT, APX, and GR activity	Mushtaq et al., 2023
*Bacillus* sp. Kz5 and *Enterobacter* sp. Kz15	*Brassica juncea*	5, and 20 mg/kg Cd	Increased Cd uptake	Increased shoot, root biomass, and photosynthetic activity	Zhang et al., 2023
*Priestia flexa*	*Oryza sativa*	25 μM As (III), and 100 μM As (V)	53.02% reduction in As (V), and 38.84% As (III) uptake	Increased shoot length, root length, fresh weight, dry weight, and chlorophyll content, but SOD, CAT, APX, and GPX activity was reduced in the plant	Majhi et al., 2023
*Pseudomonas chengduensis*			31.48% reduction in As (V), and 35.98% As (III) uptake		
*Serratia marcescens* DB1	*Oryza sativa* L.	1,000–5,000 μM Cr, and As	16.55 % reduction in Cr uptake	Increased shoot weight (19.92%), SA content (20.25%). Reduced ABA (12.71%), JA (7%), flavonoid (11.88%), polyphenol (17.58%) level. *OsMTP5, OsMTP1*, and *OsPCS1* expression decreased.	Bhatta et al., 2023
			48.9% reduction in As uptake	Shoot weight (50%), SA content (21.95%), Reduced ABA (30.30%), JA (26.58%), flavonoid (13.35%), polyphenol (25.91%) level. *OsMTP1*, and *OsPCS1* expression decreased	
*Bacillus subtilis*	*Oryza sativa* L.	15 mg/kg As	50% increased As accumulation in plants	GST, CAT, GSH activity, and total thions content was significantly decreased	Ullah et al., 2024
*Pseudomonas* sp.	Alfalfa	0.5 mM CdCL_2_	**–**	Enhanced dry weight, chlorophyll a, b, and total chlorophyll content	Chubukova et al., 2024
*Bacillus* sp.	*Oryza sativa* L.	30 ppm As	**–**	Improved vegetative parameters, chlorophyll content, GPX, CAT activity, total yield, percent grain filling, and 100 seed weight	Roy et al., 2024
*Serratia rubidaea* SR19	*Cucumis Sativus*	3, 6, and 9 ppm CdCL_2_	**–**	Increased seed germination rate (100% at 9 ppm), root, and radicle length	El-Minisy et al., 2025

^*^Indicate *Actinomycetes, Azotobacter* sp., *Azospirillum* sp., *Rhizobium* sp., *Pseudomonas* sp., *Lactobacillus* sp., *Bacillus* sp., and *Streptomyces* sp.

### 4.1 As stress

A study by (Pandey and Bhatt 2016) provided insights into the role of *Exiguobacterium* sp. As-9 strain in *Vigna radiata* growth under As stress. Results showed that As-9 inoculation significantly improved the shoot, root biomass, shoot, root length by 22.23, 39, 29.6, and 18.15%, respectively under As (V) stress. Strikingly, bacterial treatment led to a 5-fold reduction in As(V) accumulation, along with a substantial decrease in malondialdehyde (MDA) content −28.02% in shoots and 45.24% in roots—indicating mitigation of oxidative stress induced by arsenic toxicity. Moreover, (Ghosh et al. 2018) also shed light on the role of As resistant *Bacillus aryabhattai* MCC3374 (AS6) strain on reducing As phytotoxicity in *Oryza sativa*. Bacterial inoculation significantly enhanced the activity of key seed germination-associated enzymes, including amylase (51.3%) and protease (50%), as well as antioxidant enzymes such as superoxide dismutase (SOD; 27.2%), and catalase (CAT; 62.2%). Interestingly, AS6 strain also exhibited ACC-deaminase activity which is associated with the reduction of ethylene levels in rice seedlings, thereby promoting root elongation. Additionally, (Xiao et al. 2020) demonstrated the efficacy of three PGPR strains *Pseudomonas mosselii* (S6), *Bacillus thuringiensis* (S7), and *Bacillus* sp. JBS-28 (S10) on As accumulation and *Oryza sativa* growth. Notably, inoculation with strain S10 significantly reduced arsenic accumulation in brown rice by 3.50–26.01% and 9.26–10.50%, while all three PGPR strains (S6, S7, and S10) enhanced grain yield by 10.50–51.30% and 4.83–9.16% under greenhouse and field conditions, respectively. In addition to this, PGPRs application also influenced shoot, and root growth, SOD, and peroxidase (POD) activity in the presence of As.

Recently, (Pandey et al. 2023) illustrated that PGPRs consortia (*Bacillus nealsonii, Pseudomonas nitritireducens, Exiguobacterium aestuarii, Bacillus tequilensis*, and *Microbacterium paraoxydans*) significantly improved the shoot, root length, biomass, and total chlorophyll content of *Oryza sativa* L. plants. Notably, PGPRs treatment led to the downregulation of key antioxidant genes—*SOD, CAT, APX* (ascorbate peroxidase), *GST* (glutathione S-transferase), and *GPOX* (glutathione peroxidase)—suggesting a substantial reduction in oxidative stress within the plant system as compared to As (V)/As (III) treated plants. Moreover, the study by (Joshi et al. 2023) demonstrated that *Bacillus amyloliquefaciens* effectively mitigates As toxicity in *Oryza sativa* by modulating sugar metabolism and associated metabolic pathways. Metabolomic profiling revealed a significant upregulation of key metabolites involved in carbohydrate metabolism, organic acid turnover, fatty acid biosynthesis, and amino acid metabolism under As stress following bacterial inoculation. Transcriptomic analysis further indicated that the expression of key enzymes involved in glycolysis, sugar metabolism, and energy production were significantly upregulated, suggesting enhanced carbon flux. At molecular level, quantitative real-time PCR (qRT-PCR) studies result indicated that the expression of As influx, and efflux transporters like Low Silicon 1 (*Lsi1*), Low Silicon 2 (*Lsi2*), and Low Silicon 6 (*Lsi6*) were downregulated after bacterial treatment, thereby restricting As uptake and translocation. These findings highlight the potential of *B. amyloliquefaciens* in modulating metabolic and transport processes to confer As tolerance in rice.

### 4.2 Pb stress

Several studies have demonstrated the potential of Pb-tolerant PGPRs in mitigating Pb-induced phytotoxicity and enhancing plant growth under heavy metal stress. Saleem et al. (2018) demonstrated that application of Pb tolerant *Pseudomonas* strains significantly enhanced the growth performance of *Helianthus annuus* cultivated in Pb-contaminated soil (900 mg/kg). PGPR inoculation improved root length, root fresh weight, and dry weight by 28, 52, and 74%, respectively, compared to Pb stress alone. Moreover, there was a notable increase in chlorophyll a and b, carotenoid, and proline content, indicating improved photosynthetic efficiency and osmotic adjustment under stress conditions. The treatment also led to a 36% reduction in MDA levels, along with enhancements of SOD, glutathione reductase (GR), APX, and CAT activity by 26, 24, 12, and 26% respectively, suggesting a significant reduction in oxidative damage. Similarly, Abdelkrim et al. (2018) work also highlights the effect of Pb-resistant PGPR consortia on *Lathyrus sativus* growth. Results showed that PGPRs inoculation improved the plant biomass, total N, chlorophyll, carotenoid, total polyphenolic, proline, and soluble sugar content under Pb stress. The activity of ROS scavengers (SOD, CAT, APX, and GPX) were also significantly improved in Pb stress, suggesting improved antioxidant defense mechanisms. Furthermore, Pal et al. (2018) study showed that application of *Lysinibacillus varians* and *Pseudomonas putida* positively influenced growth parameters in *Capsicum annuum* L. under Pb stress. Specifically, shoot and root lengths were increased by 1.60-, 1.71-, 1.35-, and 1.15-fold, respectively, along with higher chlorophyll content, indicating enhanced photosynthetic performance. Moreover, He et al. (2020) investigated the efficacy of Pb-resistant *Bacillus* strains (QX8 and QX13) on *Solanum nigrum L*. and found a significant improvement in shoot and root dry weights by 1.36–1.96 fold, as determined by Duncan's multiple range test. Furthermore, QX8 and QX13 treatments increased shoot iron concentrations by 55 and 88%, respectively, demonstrating a positive effect on nutrient acquisition in Pb-contaminated soil. In another study, Shabaan et al. (2021) observed enhanced shoot and root lengths by 18 and 84%, respectively, in *Pisum sativum L*. inoculated with *Pseudomonas* strains under 750 mg/kg Pb stress, indicating improved biomass accumulation. Moreover, Ahmad et al. (2023) further supported these findings by showing that inoculation with *Pseudomonas* strains (S1, S2, and S3) enhanced fresh weight, dry weight, chlorophyll content, and APX activity by 36, 26, 18, and 12.24%, respectively, in *Vigna unguiculata L*. under Pb stress, reflecting improved physiological performance and antioxidative capacity.

A mechanistic insight was provided by El-Esawi et al. (2024), who demonstrated that inoculation with *Serratia liquefaciens* ZM6 significantly improved morphological, nutritional, and physiological attributes of *Glycine max L*. under 400 μM Pb stress. Enhanced levels of photosynthetic pigments, osmolytes, enzymatic (CAT, APX, POD, SOD) and non-enzymatic (AsA, GSH) antioxidants were recorded. At the molecular level, qRT-PCR results showed that ZM6 inoculation upregulated the expression of antioxidant genes (*CAT, APX, POD, Fe-SOD*) and stress-responsive genes [*chalcone synthase* (*CHS7), chalcone isomerase* (*CHI1A), phenylalanine ammonia-lyase* (*PAL*)*, isoflavone synthase* (*IFS2*)*, pyrroline-5-carboxylate synthase* (*P5CS*), and WRKY-type transcription factor (*WRKY54*)], which are critical for ROS scavenging and biosynthesis of stress-related metabolites such as proline, isoflavonoids, and flavonoids. Notably, yield-related traits including pod number, seed number per pod, and pod weight per plant were also improved. Besides this, Nie et al. (2025) also reported that co-inoculation of *Pseudomonas* and *Bacillus* species significantly enhanced plant height, biomass accumulation, root development, and antioxidant enzyme activities (SOD, CAT, POD) in *Medicago sativa L*. grown under extreme Pb concentrations (1,000 and 5,000 mg/L). Additionally, Anjum et al. (2025) further demonstrated that *Pseudomonas fluorescens* strains S5 and S10 improved shoot and root length by 18.73–65.80% and 32.77–95.79%, respectively, in *Solanum lycopersicum L*. under 500 mg/kg Pb stress. The strains also increased proline content and activities of CAT, POD, and SOD, along with a marked reduction (2.41–16.07%) in electrolyte leakage, indicating better maintenance of membrane integrity and cellular homeostasis under Pb-induced oxidative stress.

### 4.3 Cd stress

Recent studies have highlighted the critical role of PGPRs in mitigating Cd toxicity through modulation of physiological, biochemical, and molecular mechanisms in various crops. Khanna et al. (2019) reported that *Pseudomonas aeruginosa* and *Burkholderia gladioli* mitigates 0.4 mM Cd in *Lycopersicon esculentum* seedlings by downregulating the expression of *metal transporter* genes (*MT 1-16*), thereby reduced Cd uptake in the seedlings. In addition to this, Jan et al. (2019) work illustrated that the application of *Enterobacter ludwigii*, and *Exiguobacterium indicum* provides Cd tolerance to *Oryza sativa* seedlings by downregulating the expression of *OsMTP1* genes. Sahile et al. (2021) demonstrated that inoculation with *Bacillus cereus* modulates hormonal signaling in *Glycine max* under Cd stress, resulting in 23% decrease in abscisic acid (ABA) levels, and a 6–16% increase in salicylic acid (SA) content, indicative of suppression of stress-induced hormonal responses and enhanced defense signaling. Similarly, Liu et al. (2024) reported that *Bacillus siamensis* strain R27 significantly reduced Cd accumulation in *Lactuca sativa* shoots by 38.9%, while improving shoot fresh weight (29.4%), root fresh weight (40.2%), and total chlorophyll content (45.8%). Gene expression analysis revealed the downregulation of key Cd transporter genes, including *iron regulated transporter 1* (*IRT1), natural resistance-associated macrophage protein* (*Nramp1*), *heavy metal P*_1*B*_*-type ATPase* (*HMA2, HMA4), zinc regulated transporter, iron regulated transporter like protein* (*ZIP4, ZIP12)*, thereby limiting Cd uptake and translocation. In another study, Shahid et al. (2024) observed that *Rhizobium fabae* SR-22 enhanced seed germination, root and shoot length, and pigment content in *Triticum aestivum* under 2 mM Cd stress, along with increased activities of antioxidant enzymes—POD (23%), CAT (14%), and APX (34%)—suggesting an improved antioxidative defense system. *Pseudomonas geniculata*, a Cd-tolerant strain evaluated by Madhogaria et al. (2024), significantly improved growth traits of *Vigna radiata* under 80 μg/mL Cd exposure, with increases in shoot length (40 %), primary root length (45%), secondary root number (14%), and fresh weight (47%). Under 10 μg/mL Cd, total chlorophyll content rose by 58%, while Cd accumulation, H_2_O_2_ levels, and electrolyte leakage decreased by 44, 25, and 20%, respectively.

In line with these findings, AL-Huqail et al. (2025) showed that *Azospirillum brasilense* enhanced growth parameters, photosynthetic efficiency, and pigment accumulation (chlorophyll, and carotenoid) in *Oryza sativa* under 100 μM Cd stress. The bacterium also activated the ascorbate–glutathione (AsA–GSH) cycle by elevating glutathione, ascorbate, and dehydroascorbic acid levels, and significantly upregulated antioxidant genes (*SOD, POD, CAT, APX*), thereby reinforcing redox homeostasis and mitigating Cd-induced oxidative damage. Moreover, Liao et al. (2025) demonstrated that *Enterobacter hormaechei* X20 enhances Cd tolerance in *Lolium perenne* by reducing electrolyte leakage and MDA levels, indicating improved membrane stability. Under 450 mg/kg Cd stress, inoculation significantly increased uptake of Fe^2+^, Cu^2+^, Mn^2+^, and Zn^2+^, and boosted levels of amino acids, fatty acids, organic acids, and sugar alcohols, collectively contributing to improved metabolic and physiological adaptation.

### 4.4 Cr stress

Bruno et al. (2020) investigated the potential of *Bacillus cereus, Providencia rettgeri*, and *Myroides odoratimimus* in alleviating Cr stress in *Sorghum bicolor*. Bacterial inoculation resulted in significant enhancement of plant growth and antioxidant enzyme activities (SOD, CAT, and APX), while concurrently reducing the levels of stress indicators such as proline, and MDA. Molecular analysis further revealed that bacterial treatment conferred stress tolerance by modulating stress-related genes. These included antioxidant defense genes (*sod, apx1, cat*), which were upregulated, and the osmolyte regulation gene *p5cs1*, involved in proline biosynthesis was found to be downregulated, indicating a shift in stress-response modulation. In a similar context, Tirry et al. (2021) demonstrated that *Pseudomonas* sp. inoculation significantly enhanced Cr tolerance in *Medicago sativa*, reflected by substantial increases in shoot (97.6%) and root (95.4%) dry weights and a 25% increase in total chlorophyll content. This growth promotion was accompanied by a marked reduction in oxidative stress markers, including MDA (42.4%), H_2_O_2_ (59.35%), and proline (63%). Further supporting the role of beneficial microbes in Cr stress alleviation, El-Ballat et al. (2023) reported that treatment with *Azospirillum brasilense* EMCC1454 enhanced Cr tolerance in *Cicer arietinum* L. by improving morphological characteristics, photosynthetic pigments, osmolytes (proline and glycine betaine), soluble sugars, and levels of antioxidants such as CAT, APX, SOD, POD, ascorbic acid, and glutathione. Gene expression analysis under 260 μM Cr stress showed significant upregulation of key stress-responsive genes, including *CAT, SOD, APX, CHS, dehydration-responsive element-binding protein 2A* (*DREB2A*), *CHI*, and *PAL*, indicating the activation of both enzymatic antioxidant defense and stress signaling pathways.

Similarly, Naz et al. (2023) evaluated the effect of *Mesorhizobium* RC3 on *Cicer arietinum* L. under Cr stress and observed enhanced shoot and root lengths by 12.38 and 10.87%, respectively, along with increased nodule number (6.64%) and dry nodule weight (13.77%). Physiological and yield-related attributes such as chlorophyll content (6.83%), leghaemoglobin (9.47%), protein content (16.83%), and seed yield (27.45%) were also significantly improved in inoculated plants compared to uninoculated controls. In addition, Mohanty and Mohapatra (2023) emphasized the synergistic potential of co-inoculating PGPR (*Rhizobium* sp. and *Bacillus* sp.) with phosphate-solubilizing bacteria (PSB) (*Microbacterium* and *Pseudomonas* sp.) in *Vigna radiata* L. exposed to 100 ppm Cr stress. The combined microbial treatment significantly improved key growth metrics, including shoot and root length, leaf number, leaf area, and total chlorophyll content, highlighting the advantage of microbial consortia over single inoculants in mitigating Cr-induced phytotoxic effects.

**Figure 3:**
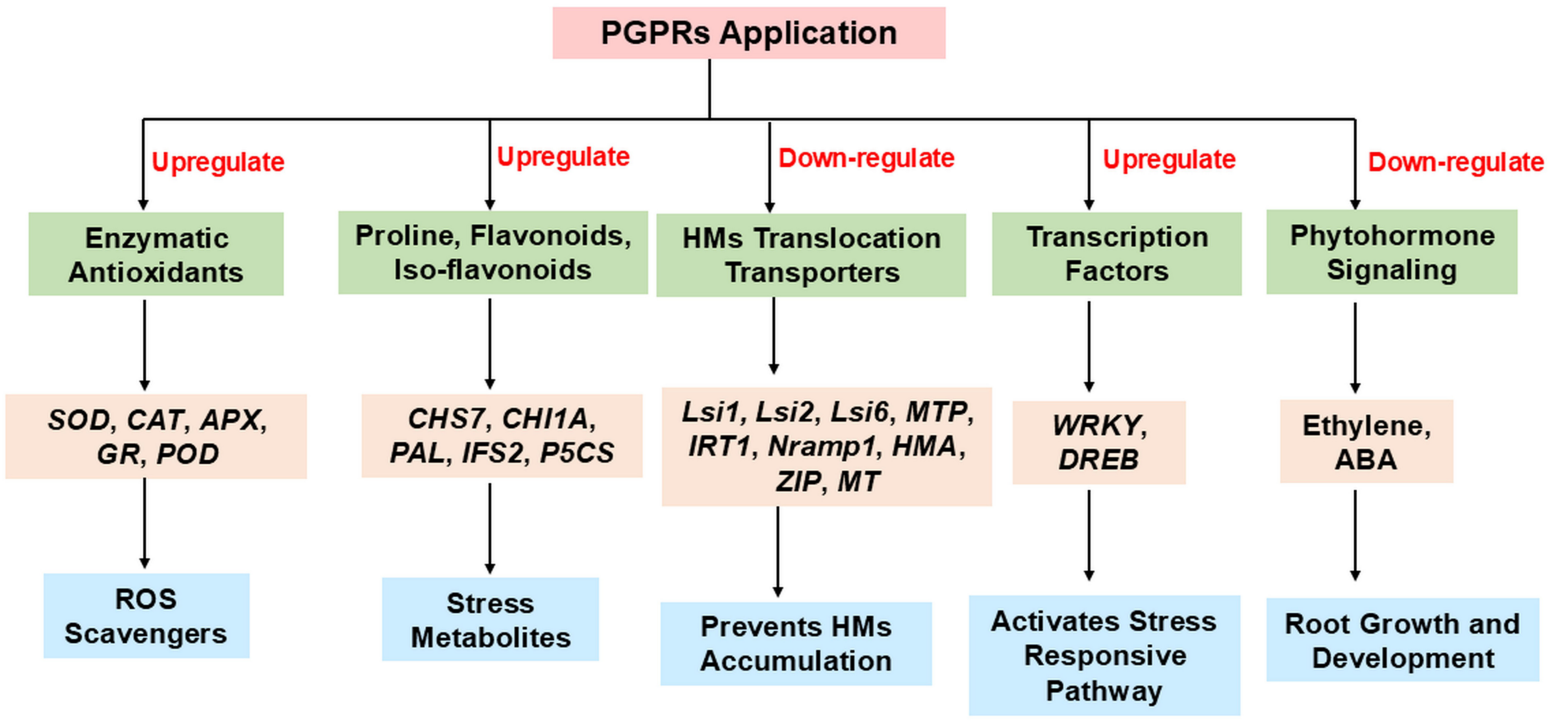
Overview of molecular pathways targeted by PGPRs in plants for providing HMs stress resilience.

Overall, HMs tolerant PGPRs effectively mitigates metal-induced phytotoxicity by enhancing plant growth, physiological performance, regulating osmolytes accumulation, modulating antioxidant defense, and stress-responsive gene expression, offering a sustainable strategy for stress amelioration. These effects are summarized in the Figure 3.

## 5 Conclusion and future roadmap

The increasing contamination of agricultural soils with HMs represents a critical threat to environmental quality, agricultural sustainability, and human health. This review synthesizes current knowledge on the mechanisms of HMs mitigation by PGPRs, their role in improving plant growth through enhanced nutrient acquisition, phytohormone production, stress alleviation, and modulation of soil physicochemical properties. However, despite encouraging laboratory results, the widespread application of PGPR-based remediation in real-world agricultural systems remains limited, primarily due to a lack of mechanistic insights, field-scale validation, and scalable deployment strategies. Bridging the lab-to-field gap is essential to enable effective translation of research outcomes into real-world applications. Therefore, future research must focus on conducting long-term, multi-location field trials to evaluate the consistency and adaptability of PGPRs under diverse environmental conditions. The integration of multi-omics approaches such as genomics, transcriptomics, metabolomics, and proteomics can help unravel the molecular and biochemical pathways underlying HMs detoxification and PGPR–plant interactions. In addition, the development of synthetic microbial consortia with complementary functional traits could offer synergistic advantages over single-strain inoculants. Exploring plant genotype-specific responses to PGPRs will further enable tailored bioremediation strategies suited to different crops and soil types. Moreover, long-term ecological monitoring is required to understand the impact of PGPR introduction on native microbial communities and overall soil health.

Bridging the gap between laboratory research and field application through interdisciplinary efforts will be key to unlocking the full potential of PGPRs in advancing climate-resilient and sustainable agriculture. With proper scientific validation and policy support, microbial bioremediation can serve as a cornerstone technology for ensuring food security and environmental sustainability in the 21st century and beyond.
